# Arsenicum-album

**Published:** 1895-10

**Authors:** 


### HOMŒOPATHIC PHYSICIAN,

A MONTHLY JOURNAL OF

HOMŒOPATHIC MATERIA MEDICA AND CLINICAL MEDICINE.

"If our school ever gives up the strict inductive method of Hahnemann, we are lost, and deserve only to be mentioned as a caricature in the history of medicine."—CONSTANTINE HERING.

Vol. XV. OCTOBER, 1895. No. 10.

### EDITORIAL.

ARSENICUM-ALBUM.—Continuing the notes from last month, we may remark that the swelling of the face mentioned in the pathogenesis of Arsenicum occurs especially about the eyelids, and is generally observed in the morning. Kali-carbonicum has bag-like swelling between upper eyelids and the eyebrows. This, said Dr. Lippe, is Bœnninghausen's key-note for Kali-carbonicum in whooping cough. Phosphorus has œdematous swelling of the face under the eyes.

Apis also has œdematous swelling under the eyes together with swelling of the lids.

Iodine has œdematous swelling of the eyelids.

The Arsenic patient has sunken face with waxy appearance. It has, also, bluish discoloration of the face. The face is marked with ulcers at times. The Arsenic patient has very dry lips producing a brown streak along the middle of the vermilion border, especially of the under lip. There are also purple black spots on the lips.

Arsenic has sensation of a hair upon the tongue. This is similar to Silicea. Kali-bichromicum has sensation of a hair on the back part of the tongue not relieved by eating or drinking.

Sulphur has sensation of a hair in the throat.

Valerian has nausea from sensation of a hair in the throat.

Kali-bichromicum has tickling high up in the left nostril as if from a hair located there.

Hydrastis, tickling as if from a hair in the right nostril.

Arsenic has great desire for rum.

In Bœnninghausen's *Therapeutic Pocket Book*, edition of 1847, at pages 61, 62, 63, and 64, is given a list of the desires and aversions of the principal homœopathic remedies. In Dr. T. F. Allen's edition of the same author there is a similar list, to which the editor has added a number of remedies proved since Bœnninghausen's time. This list may be found in Allen's edition, at page 67.

This list should always be consulted in making a prescription as it often leads up to the right remedy.

The vomiting of Arsenicum is variously colored—brown, black, red (from blood), and green. This peculiar vomiting should be compared with Apis, Croton-tiglium, and Veratrum.

The vomiting of Arsenic is frequently accompanied with diarrhœa.

Vomiting followed by diarrhœa is an indication for Arsenicum. Vomiting and diarrhœa occurring together is Dr. Guernsey's key-note for Arsenicum.

Vomiting from drinking water is also another of Dr. Guernsey's key-notes for Arsenicum.

In connection with these symptoms of Arsenicum, Dr. Lippe once related a case of a brilliant cure which he made with Arsenicum. It was a case of diarrhœa. There was severe pain in the bowels from drinking the slightest quantity of water, followed by vomiting. The abdomen was so sensitive the patient could not bear the slightest pressure of the sheet, and there was intense restlessness. Arsenicum was given and the symptoms all subsided, and the patient quickly got well.

Phosphorus has vomiting after drinking water as soon as the water gets warm in the stomach. This is Dr. Guernsey's key-note and has been mentioned before.

The editor takes the liberty of adding the following symptoms of vomiting :

Aconite, vomiting after drinking water. The patient declares he will die, then drinks and vomits again. This symptom also has been mentioned before.

Silicea, vomiting after drinking water. The water tastes badly.

Creosote, vomiting after greedy drinking.

Baptisia, vomiting of fluids but not of solids.

Æthusia, vomiting of milk soon after it is swallowed.

Antimonium-crudum, vomiting of what has been eaten and drunk.

Sulphuric-acid, vomiting of food in mouthfuls.

Arnica, vomiting of the least food.

Apis, Arsenicum, Bryonia, Digitalis, Ferrum, Veratrum, and Zinc, all have vomiting after eating.

Stannum, vomiting of water from the smell of food.

Sarsaparilla, vomiting from thinking of food.

Graphites, vomiting and purging with icy-cold sweat and headache. This is similar to Veratrum.

Veratrum has vomiting whenever he moves or drinks.

Veratrum has bitter vomiting and Camphor has sour vomiting.

Æthusia, vomiting with convulsions, eyes turned downward, thumbs clenched in the palms of the hands or sometimes turned backward.

Ailanthus, vomiting on sitting up.

Antimonium-tartaricum (Tartar-emetic), violent straining to vomit, with perspiration on the forehead.

Antimonium-crudum, frightful vomiting; persistent vomiting which nothing can stop. Vomiting with convulsions. This is Dr. Guernsey's key-note.

Nux-vomica, periodical attacks of vomiting.

Arsenic, vomits food the instant it enters the stomach, but Nux-vomica, vomits it some hours after eating.

Pulsatilla, vomiting after each meal, preceded by pressure in the pit of the stomach.

Many more of these indications for vomiting might be added, but they would hardly be valuable unless properly classified.

This has not yet been done, and they stand in the editor's note-book in some disorder, as they have accumulated gradually through several years of experience.

